# An open-source framework for large-scale, flexible evaluation of biomedical text mining systems

**DOI:** 10.1186/1747-5333-3-1

**Published:** 2008-01-29

**Authors:** William A Baumgartner, K Bretonnel Cohen, Lawrence Hunter

**Affiliations:** 1 Center for Computational Pharmacology, University of Colorado School of Medicine, Aurora, CO, USA

## Abstract

**Background:**

Improved evaluation methodologies have been identified as a necessary prerequisite to the improvement of text mining theory and practice. This paper presents a publicly available framework that facilitates thorough, structured, and large-scale evaluations of text mining technologies. The extensibility of this framework and its ability to uncover system-wide characteristics by analyzing component parts as well as its usefulness for facilitating third-party application integration are demonstrated through examples in the biomedical domain.

**Results:**

Our evaluation framework was assembled using the Unstructured Information Management Architecture. It was used to analyze a set of gene mention identification systems involving 225 combinations of system, evaluation corpus, and correctness measure. Interactions between all three were found to affect the relative rankings of the systems. A second experiment evaluated gene normalization system performance using as input 4,097 combinations of gene mention systems and gene mention system-combining strategies. Gene mention system recall is shown to affect gene normalization system performance much more than does gene mention system precision, and high gene normalization performance is shown to be achievable with remarkably low levels of gene mention system precision.

**Conclusion:**

The software presented in this paper demonstrates the potential for novel discovery resulting from the structured evaluation of biomedical language processing systems, as well as the usefulness of such an evaluation framework for promoting collaboration between developers of biomedical language processing technologies. The code base is available as part of the BioNLP UIMA Component Repository on SourceForge.net.

## Background

This paper investigates the hypothesis that structured evaluations are a valuable addition to the current paradigm for performance testing of large language processing systems. Support for the claim that thorough, structured evaluations are a prerequisite for further advances in the field of text mining has recently come from a surprising corner. In a recent keynote speech at the 10th annual meeting of the Conference on Natural Language Learning (CoNLL), Walter Daelemans, a noted proponent of machine-learning-based approaches to natural language processing (NLP), pointed out that the machine learning community is falling short of its potential to ask and to answer interesting and important questions not just about machine learning techniques, but about the light that machine learning can shed on theoretical issues as well. Daelemans points out that evaluations of machine learning algorithms often produce deceptive or incomplete results due to ignoring the complex interactions that characterize both language and language processing tasks on one hand, and machine learning algorithms on the other. Some of these interactions are related to aspects of machine learning systems specifically, such as interactions between algorithm parameters and sample selection or between algorithm parameters and feature selection. Other interactions come from data – interactions between training set contents and training set size, or between training set and external knowledge sources.

Conducting better evaluations, then, requires complex comparisons involving many alternative combinations of software and data. This requires a framework that can support activities requiring complex combinations of applications that can be connected in flexible and configurable workflows, as well as the ability to import, store, query, reformat, and share a tremendous diversity of data types. We have built an extensive code base that facilitates performing exactly these functions for language processing in general, and biomedical language processing in particular. This code base has been made available under an open source license in the BioNLP UIMA Component Repository on SourceForge.net [1] [see also Additional file 1].

The system uses UIMA [2-4], the open source Unstructured Information Management Architecture, as its infrastructure. UIMA is a robust data management framework for processing, or "annotating," unstructured data, the prototypical example of which is free text. The essence of our use of UIMA is as a middleware layer that facilitates the smooth interaction of many different NLP tools that were not originally designed to interoperate with each other. The UIMA paradigm necessitates the use of a standardized interface, thus ensuring stable data transfer among system components. It should be noted that the use of UIMA in general is not limited to text processing applications. It is intended for use with any type of unstructured data, e.g. images, audio, video, etc. Our discussion of UIMA, however, will focus on its use in the text processing domain.

The UIMA framework is well-suited for the construction of document processing pipelines. There are three basic component types used in a UIMA pipeline. The *Collection Reader *component acts as an input device for the pipeline. The collection reader instantiates the data structure that is shared by the different UIMA components, known as the *common analysis structure (CAS)*, and initializes it with the document text. The CAS is a flexible data structure capable of storing not only the document text, but annotations of the text, as well as metadata. We define an annotation simply as a pair of character offsets into the original document text associated with a specific semantic type. The pair of character offsets is said to define the *span *of text covered by the annotation. Typically, a separate CAS is generated for each document that is processed.

Once initialized, the CAS is sent down the processing pipeline. Components that act on the contents of the CAS, and in particular, those that add content to the CAS, are known as *Analysis Engines*. Analysis engines come in two forms: *primitive *and *aggregate*. An example of a primitive analysis engine would be a tokenizer, which takes raw text as its input and produces as output a set of annotations that describe token boundaries. Aggregate analysis engines consist of combinations of primitive analysis engines where downstream analysis engines may rely on annotations created during upstream processing. An example of an aggregate analysis engine would be a part-of-speech tagger that uses token annotations created by a tokenizer as its input and adds part-of-speech tags to the tokens.

The third major component in a UIMA pipeline is termed the *CAS Consumer*. CAS consumers are similar to analysis engines in that they act on the contents of the CAS. They do not, however, add content to the CAS. CAS consumers represent the end of the pipeline. An example CAS consumer, particularly relevant to this paper, would be an evaluation platform that compares annotations added to the CAS by an upstream named entity recognizer analysis engine to a set of predefined gold standard annotations.

The semantic types for annotations generated during processing are specified through the creation of a UIMA *Type System*. The type system facilitates inheritance of annotation types, as well as specifications for metadata. The released version of our evaluation platform uses a general text annotation class to represent all semantic types. Further details of our type system are available in the evaluation platform documentation [1].

There are several advantages to using a framework such as UIMA for NLP system evaluation. The common interface for passing data among system components all but removes the need to write custom code for stitching together various text processing modules. The ramifications of this are two-fold. In terms of constructing code, the text processing machinery can be isolated from the communications and data transfer mechanisms. This promotes more modular code and functional testing at the individual component level. Furthermore, not only does the use of a standardized interface among components enable various tools that were not originally designed to be used in concert, it promotes the sharing of such components among developers, and perhaps more importantly, among the NLP community. Recently, several publicly available repositories for NLP tools integrated into the UIMA framework have been created online: the Tsujii Lab UIMA Repository [5], the JULIE lab tools page [6], the CMU UIMA Component Repository [7], the BioNLP UIMA Component Repository [1], and the UIMA Sandbox [8]. Given that the UIMA paradigm necessitates the use of a standardized interface, thus ensuring stable data transfer among components, the framework enables users to organize disparate tools into complex interconnecting workflows. It should be noted that availability of source code is not a prerequisite for integrating an application into UIMA. Access to the source code can make the transition easier, however the only real requirement is access to a working implementation of the application to be integrated. The use of this infrastructure in combination with our code base makes plausible the large-scale evaluation of NLP systems for which we see a need.

We demonstrate the capabilities of the evaluation platform through two experiments. The first experiment involves an intrinsic evaluation: we examine the performance of nine gene name taggers on five evaluation corpora using five different definitions of correctness. The second experiment involves an extrinsic evaluation: we examine the effects of the different gene name taggers and three different methods for combining their outputs on a subsequent task – gene normalization.

Gene mention (GM) identification is a classic named entity recognition problem in the biomedical natural language processing (BioNLP) field, and one that has been studied extensively [9,10]. The task of gene mention identification is to detect where gene names appear in text. For example, given the following input text: *p53 induces monocytic differentiation*... [PubMedID:17309603], a gene tagger should detect the gene name *p53*, and (optionally) that it starts at character 0 and ends at character 3. The difficulty in identifying gene mentions in biomedical text stems from a number of factors. First, there is no standard nomenclature for naming genes or distinguishing between genes and gene products (proteins). The latter issue is typically ignored, treating gene names and protein names mentioned in text as equals. For the former, the yeast community is one exception. Its systematic gene names typically begin with *Y *and encode information such as where the gene is located in the yeast genome [11], e.g. *YAL001C*, which corresponds to the first open reading frame to the left of the centromere on chromosome I. Many Drosophila genes are particularly difficult to recognize automatically in text, e.g. *a *[EntrezGene:43852], *lush *[EntrezGene:40136], and *van gogh *[EntrezGene:35922]. Ambiguities exists among genes and other entity types as well, e.g. the gene *corneal endothelial dystrophy 1 *[EntrezGene:8197] has official symbol *CHED1 *and alias symbol *CHED*, while the abbreviation *CHED *is also used in the literature to refer to a specific cell type, the *Chinese hamster embryonic diploid cell line *[PubMed:2398816]. Various approaches to solving this problem have been attempted, ranging from trying to match text to a list of known gene names (the dictionary approach) to using machine learning techniques to create a statistical model that can be used to identify genes in text. The dictionary approach has the obvious disadvantage of being unable to identify a gene that is not explicitly mentioned in the dictionary, and thus is potentially out-of-date from the moment the dictionary is created, while the machine learning approach must rely on a training corpus that is typically expensive to generate. The machine learning approach has generally been shown to out-perform the dictionary approach [9,10].

Comparing GM systems via the published literature is often difficult because they are evaluated on different corpora, modified corpora, or worse, proprietary corpora, thus making impossible direct comparison with other published systems. A further complication, as Daelemans points out, is the all too frequent unfairness seen in the literature when optimized systems are compared to systems using their default configuration. This difficulty motivated the creation of the BioCreative [9,10]. and JNLPBA [12] shared tasks.

For some applications, e.g. detecting the presence of a statement about protein-protein interaction, having output from a GM system, i.e. knowing that a gene mention is present, may be sufficient. The usefulness of GM system output, however, will not be realized to its utmost until the output can be reliably grounded to an external resource, such as a database. The task of gene normalization (GN) addresses this issue by linking a gene name mentioned in text to a specific gene identifier in a database. For example, using our sample text from the GM task: *p53 induces monocytic diffierentiation*... [PubMed:17309603], the output of a GN system should provide a link from [EntrezGene:7157] (assuming the text is discussing the *Homo sapiens *p53 gene) to the entire text string, or preferably to the text *p53 *itself. Approaches to the GN task have varied. Some work directly on the input text itself, while others use GM systems to identify potential genes and then try to normalize the gene mentions that were found. The latter approach has the advantage of being able to know where exactly in the text a particular gene is being discussed. This knowledge aids in further extraction tasks, such as determining the relationship between a pair of gene mentions. Some of the difficulties in the GN task, as in the GM task, also lie in ambiguity among gene names. The ambiguity from the GN perspective, however, is not between gene names and other entity types, but rather between the gene names themselves. There are numerous examples of species ambiguity among gene names, i.e. two or more species sharing the same gene name. For example, *cdc2l5 *is used as a gene symbol for *cell division cycle 2-like 5 *in both human [EntrezGene:8621] and in mouse [EntrezGene:69562]. There are also examples of gene name ambiguity found in a single species, i.e. two different genes sharing the same name or symbol. The human gene *corneal endothelial dystrophy 1 *[EntrezGene:8197] has official symbol *CHED1*, and alias symbol *CHED*, while human *cell division cycle 2-like 5 *[EntrezGene:8621] also has *CHED *as an alias symbol.

In recent years, several community-wide evaluations [9,10,12] have addressed these issues, yielding valuable insight into some of the factors that affect both GM and GN performance. Nonetheless, they have left many issues unexplored, and we will show that their results are not sufficient to provide a nuanced understanding of GM and GN systems.

It will be seen that this work has relevance both to the nature of discovery in biomedical text mining and to the facilitation of collaboration in the BioNLP field. Structured evaluation has not generally been practiced by the text mining community; we present here a novel and surprising discovery about the interaction between gene mention detection and gene normalization for one GN system and about the high tolerance of this gene normalization system for low gene mention system precision. This would not be supportable without performing the sort of structured evaluation described here. Furthermore, the particular evaluation performed would have been *possible *without the availability of an architecture like the one described here, but it would not have been *practical*, due both to the scale of the evaluation – it involved 4,097 different configurations of tools and algorithms – and to the technical issues involved in coordinating the input requirements and output formats of nine different gene mention recognition systems. We return to the relevance of this work to scientific collaboration in the Conclusion.

## Implementation

### Evaluation methodology

Appropriate scoring of the output of information extraction systems in general, and BioNLP systems in particular, is not a straightforward proposition [13]. There are many ways to classify the matching criteria. Our system uses a variety of comparison metrics described by Olsson et al. [13] for scoring annotations. The code itself is modular in construction, promoting extensibility and ease of incorporation of other comparison metrics.

A UIMA wrapper was created for each of the tools and resources that we used in the evaluation – nine gene taggers, three methods for combining GM system output, a GN system, and an evaluation platform with five scoring measures – enabling them to interact with the UIMA framework. Collection readers for each of five GM evaluation corpora and the BioCreative II GN task data set capable of extracting the gold standard annotations and original document text from the various evaluation corpora were also constructed. Comparison of all of the tools was conducted in parallel by plugging each into the evaluation system. The evaluation component, which exists as a CAS consumer, computes the precision, recall, and F-measure for each upstream analysis engine by comparing the results to those pre-defined in the evaluation corpora.

### Evaluating a collection of named entity recognizers

We demonstrate the scalability and versatility of our evaluation platform through the evaluation of multiple GM systems (gene taggers) using multiple biomedical corpora and multiple evaluation metrics. For this demonstration, we evaluated nine gene taggers on five biomedical corpora that have been manually annotated for gene and/or protein names. The gene taggers are evaluated in parallel, with each evaluation corpus requiring a separate run. All taggers were used "out-of-the-box" – no optimization was performed for any of the taggers during the evaluation.

The experiments reported here required using many GM tools, which were generously made available by their creators. Some performed much better, and some much worse, than others. The aim of this paper is to demonstrate the utility of our evaluation system and its ability to handle large-scale complex evaluations that would otherwise be prohibitive to conduct. For this reason, we do not identify the resulting scores with the systems that produced them. Furthermore, we did not re-train the machine-learning-based methods on the test corpora, choosing instead to use the tools as they are provided out-of-the-box. Our motivation for this is two-fold. First, the focus of this paper is the framework that we are introducing for evaluating NLP tools and not the performances of the individual tools. The tools merely serve to provide a use-case for this system. Second, we feel that using tools out-of-the-box is an accurate depiction of how the tools are typically used. Since many of them require a training corpus to retrain, and since training corpora are expensive to create, we assume that they are commonly used as they are distributed. This assumption is based mainly on our use of the tools in the past and on published descriptions of uses of such tools. Although we have generally not identified specific systems here, for purposes of reproducibility we list the publicly available GM systems that were used to demonstrate the evaluation platform: AbGene [14], ABNER [15], GeneTaggerCRF [16], KeX [17], LingPipe [18], and the Penn BioTagger [19]. Two other gene taggers that are not currently publicly available were also used: the CCP gene tagger [20], and a dictionary-based tagger built using gene names from the Entrez Gene database.

The five corpora used to evaluate the GM systems were chosen based on public availability and broadness of scope and size. The corpora used were the Bio1 corpus [21,22] (100 documents); the PennBioIE oncology corpus [23,24], consisting of 1158 abstracts about molecular genetics of oncology; the iProLink corpus [25,26] annotated for proteins using two sets of annotation guidelines over the identical set of 300 abstracts; the Texas corpus [27,28], consisting of 750 Medline articles containing the word "human;" and the Yapex corpus [29-31], composed of 99 abstracts resulting from a query requiring the "protein binding" MeSH term and the words "interaction" and "molecular." All five corpora are comprised of titles and abstracts of biomedical articles.

### Evaluating a complex BioNLP system

The interplay between components in BioNLP systems can be critical, and is often unexplored fully due to the difficulty of testing the many potential component combinations. Using a structured data management architecture in conjunction with the evaluation system under discussion addresses many of these issues inherently. We have taken advantage of the nature of our system to conduct an evaluation that would otherwise be challenging both in terms of creating the various combinations of components and in terms of keeping track of the output.

The test case for this more complex evaluation is a gene name normalization system [32] constructed for the 2006 BioCreative Gene Name Normalization task [33]. The GN system used in this example relies on gene annotations as input, and we will use many of the components generated for the gene tagger evaluation discussed in the previous section to produce these annotations. The GN system evaluated is discussed in detail in Baumgartner et al. [32]; here we provide a brief synopsis of its design.

The basic methodology of the GN system is a dictionary matching approach. A lexicon of gene names was created using the gene names and synonyms found in the Entrez Gene database [34]. Each gene name and synonym underwent a regularizing procedure that removed punctuation, converted Roman numerals to Arabic numerals, and converted Greek symbols to single characters, among other things. Gene mentions identified by the GM systems were regularized in an identical manner after a conjunction resolution step. Exact string matching was used to link gene mentions and the gene lexicon. If a gene mention matched to more than a single lexicon entry a disambiguation procedure was performed.

This GN component is complex in itself, having multiple parameters that can be adjusted. For the purposes of this demonstration and to increase the clarity of our output, we fixed the parameter settings on the GN system and varied the selection of gene taggers only. The same collection of nine gene name taggers was used as input to the GN system [14-20] as were evaluated in the previous section.

Two different analysis engines were constructed for combining the results of the gene taggers prior to GN input. Combining gene tagger results is not crucial to the GN task if we are only interested in document-level annotations (as we are in this case). We have previously shown, however, that it is possible to increase aggregate tagger performance by combining gene tagger output [32]. The overlapping-mention-resolving component aims to maximize recall by keeping all gene annotations, but resolving those that overlap. When an overlap between two gene mentions is detected the gene mention with the longer span is kept, and the other discarded.

The second analysis engine created for combining gene tagger output is a consensus filter. The consensus filter is analogous to a voting scheme. Each tagger votes, and a gene annotation is kept if it accumulates a certain threshold of votes. If the threshold is not met, the gene mention is removed from the gene tagger output. The only constraint on the threshold is that it must be greater than one and less than or equal to the number of gene taggers being used. For simplicity, each tagger is weighted equally in this analysis. The combination of the consensus filter followed by the overlapping filter is also an option that is explored. Given that we have nine gene taggers and the choice of one of three filters plus the variable consensus threshold, there are 4,097 different possible combinations to explore. It is important to note that although this GN system is somewhat complex, it is actually quite simple when compared to some other BioNLP systems; an information extraction system for the BioCreative protein-protein interaction task [35] would likely include components for GM, GN, relation extraction, and many lower-level processing tasks, such as sentence segmentation, tokenization, etc., *all *of which potentially interact in unexpected ways.

The gold standard for this experiment was the training data from the BioCreative 2006 GN task. The data set consists of 281 titles and abstracts. Gene names and associated Entrez Gene identifiers are located in a separate file. The BioCreative task was evaluated on a document-level basis, and our evaluation system will do the same. To avoid the complication of determining species the corpus was intentionally designed and annotated with only human genes.

With the goal of quantifying the relationship between the quality of input to the GN system and GN system performance as a whole, all 4,097 system combinations were tested. All combinations were run over the course of two days on a single workstation (Linux, dual 2.8 GHz Xeon processors, 2 GB RAM).

## Results

### Evaluating named-entity recognition systems against different corpora with varying match criteria

**Figure 1:**
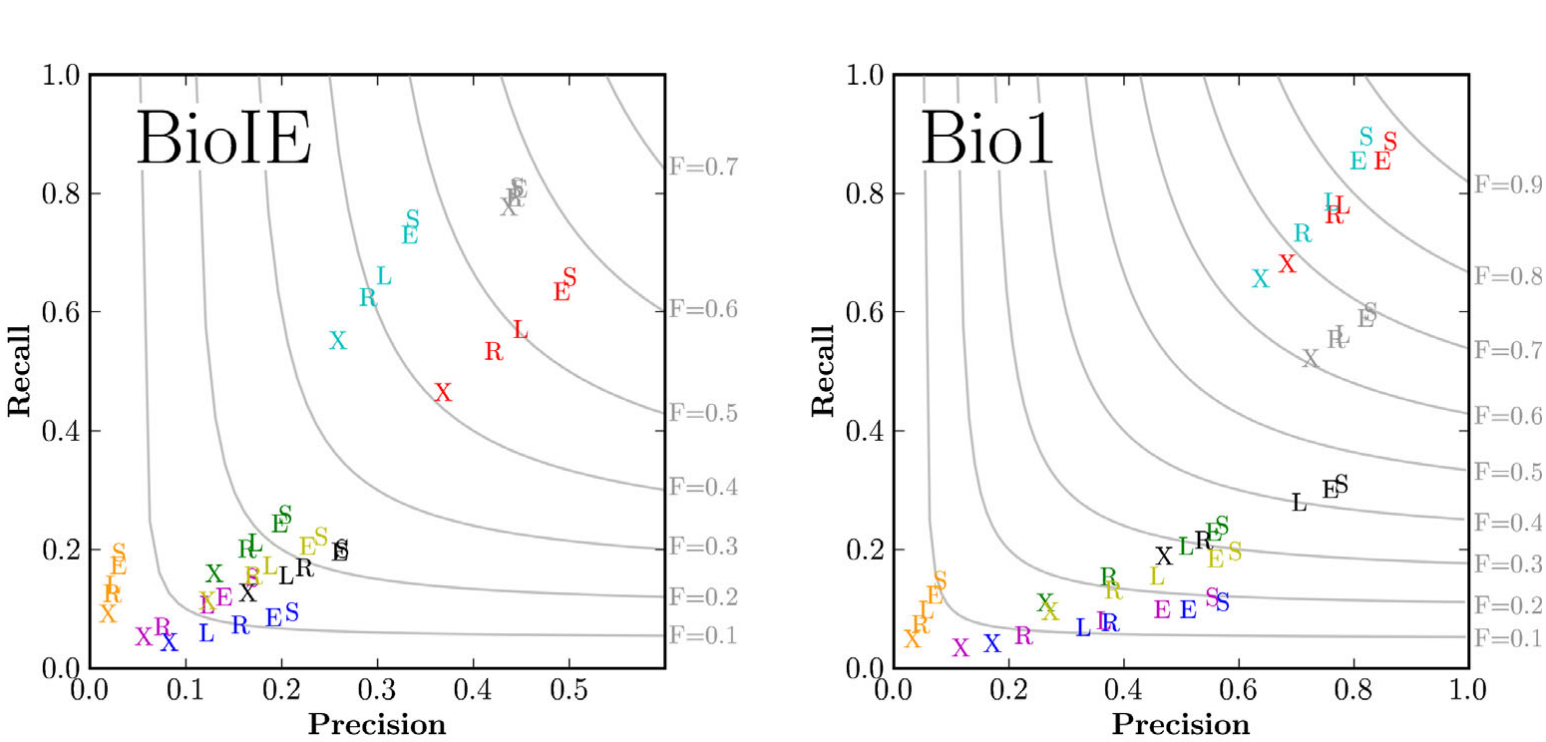
**GM system evaluation**. Evaluation results for nine gene taggers are shown for two of the five corpora used (PennBioIE Oncology, left; Bio1, right). There are 45 data points in each graph. Five evaluation metrics – *X*, Strict: spans must match exactly; *S*, Sloppy: spans must overlap; *L*, LeftMatch: span starts must match; *R*, RightMatch: span ends must match; *E*, EitherMatch: span start or end must match – were used to evaluate each tagger. Different colors are used to distinguish between the taggers. F-measure contour lines are displayed in gray, with the corresponding value listed on the right, also in gray.

The corpus used to test a language processing system is a critical decision, as is evident from the results of the gene tagger evaluation (Figure 1). The nine taggers evaluated are distinguished by color in Figure 1. Although intra-tagger trends appear consistent when comparing among corpora – the best performance is seen with the Sloppy match criterion (S), followed by the EitherMatch (E) criterion, then either the LeftMatch (L) or RightMatch (R) criterion, and finally the Strict (X) criterion – overall tagger performance can change substantially. Note the differences in the precision scales for the two graphs – performance is dramatically reduced overall in the PennBioIE Oncology corpus [23] (left) when compared to the Bio1 corpus [22] (right). Further, relative tagger performance can vary depending on the test corpus. Note the separation between the *red *and *cyan *taggers when evaluating on the PennBioIE corpus that is not evident when using the Bio1 corpus, as well as the decrease of the *gray *tagger performance relative to the *red *tagger when using the Bio1 corpus. The patterns seen in Figure 1 illustrate the importance of corpus selection. Each corpus used was developed by a different research group, and potentially for a different purpose. The annotation guidelines used during corpus construction shape the end result, and it is likely that each corpus has a slightly different definition for marking up genes and proteins. Determining the differences among these corpora that result in the observed gene tagger performance differences is non-trivial and is not addressed in this paper. It is clear from Figure 1, however, that evaluation corpus selection can influence gene tagger performance greatly. Consequently, the performance of a system on a single evaluation corpus probably should not be generalized to its performance on other evaluation corpora.

### Downstream consequences of lower-level processing: effect of gene tagging choice on GN performance

**Figure 2:**
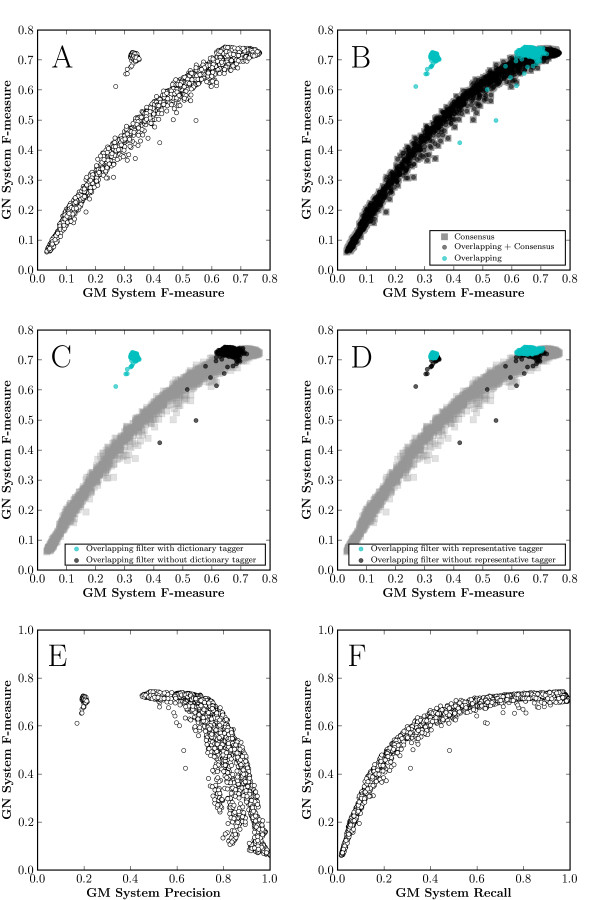
**GN system evaluation**. Results from the GN system evaluation. **(A) **GN system performance (F-measure) as it relates to the combined gene tagger performance. **(B) **GN system performance based on each of the three methods for combining gene tagger output (Overlapping, Consensus, Consensus followed by Overlapping). **(C) **GN System performance highlighting the combination of the overlapping filter with and without use of the dictionary-based GM system. Data points generated using the other filters are shown in gray. **(D) **Same as C, with the presence/absence of another representative tagger shown. **(E) **and **(F)**: GN system performance as it relates to combined gene tagger precision and recall, respectively.

Results from 4,097 unique combinations of gene taggers and the three combining approaches were generated. Figure 2A shows the performance of the GN system relative to the performance of the combined gene taggers. As might be expected, a definite correlation between gene tagger performance and gene normalization system performance exists (Pearson's correlation coefficient = 0.917, p < 0.0001). Interestingly, however, the graph is not as uniform as might be expected. For example, note the cluster of data points detached from the main curve with increased GN system performance at a lower gene tagger performance.

Plotting performance with regards to the three different gene tagger combination methods (Figure 2B) provides a clue as to the nature of this island of data points – each point in the island is associated with use of the overlapping filter. Other points generated using the overlapping filter can be seen in the main curve, however, so use of the overlapping filter cannot be the sole explanation for the observed clustering. The rest of our analysis focusses on the performances when the overlapping filter was used (Figures 2C and 2D). When we label the points based on the presence or absence of the individual gene taggers, further information is revealed. Figure 2C shows that the isolated grouping of data points includes only gene tagging systems that used the dictionary-based gene tagger, and that none of the points on the main curve were generated from systems using the dictionary-based tagger in conjunction with the overlapping filter. Figure 2D is a representative plot of one of the other eight taggers, showing a mixture of presence and absence in both the main curve and isolated cluster when using the overlapping filter. Figure 2D provides further evidence that the presence of the dictionary-based tagger plays a role in the island of data points away from the main curve; the isolated data points are a result of the combination of gene tagging systems that use the overlapping filter in conjunction with the dictionary-based tagger. As dictionary matching has been shown to favor recall over precision (Figure 2 in [36]), and the overlapping filter is geared towards preserving recall by keeping all gene mentions, we hypothesize that this island of points suggests that the performance of the GN system under test is influenced greatly by the gene tagger recall, and less so by gene tagger precision. This hypothesis is confirmed when we plot GN system performance relative to the gene tagger precision and recall in Figures 2E and 2F, respectively. Figure 2E demonstrates a negative correlation between gene tagger precision and GN system performance (Pearson's correlation coefficient = -0.7103, p < 0.0001), while Figure 2F shows a strong positive correlation between gene tagger recall and GN system performance (Pearson's correlation coefficient = 0.8725, p < 0.0001).

From this analysis, we can conclude that the performance of the GN system tested here is largely reflective of the combined gene tagger recall and less dependent on how the gene tagger system performs overall (i.e. as reflected by the F-measure, which also takes precision into account). Although there initially appeared to be a straightforward relationship between GN system performance and overall gene tagger performance (Figure 2A), our structured evaluation has given us a more nuanced understanding of the relation between GM performance and GN performance. This finding suggests that the GN system itself is filtering out false positive gene mentions to a large degree, a previously unknown characteristic of this system, and one that can be leveraged in future GN system development. It is this inherent filtering that is responsible for increased overall performance with reduced precision on the input GM data. With this new insight, gene tagging systems that were previously avoided due to their mediocre performance levels in terms of F-measure can now be added to the system, as long as their recall is relatively high.

## Discussion

The language processing community has a long history of concern with evaluation [37], and evaluation remains an ongoing focus of the community through competitive evaluations and focused conferences [38]. While recognizing that these shared tasks have been highly beneficial for the field, there are at least two reasons that they do not produce as much insight as they could. First, the competitions tend to conflate team-specific factors (e.g. limits in computational or labor resources) with the performance of the approach that a team used. While good performance in a competition is clearly indicative of merit, poorer performance may be more indicative of some confounding factor than of a lack of technical innovation or insight. A related concern is the narrowing over time of the tools and techniques used. In pursuit of high performance, many teams try minor variations on the winning formula from the previous year, rather than working to ensure that a broad diversity of tools and approaches is being evaluated.

While the shared task paradigm gives clear data on the state of the art in a particular task and whether it has advanced from year to year, it provides much less detailed information about why a certain system did well (or poorly) and which aspects of a system are the limiting factors that deserve research attention. Hirschman and Thompson [39] contrast *performance evaluation*, which compares multiple programs to each other in terms of some metric, and *diagnostic evaluation*, a systematic exploration of performance of one or more programs with respect to some problem space. Cohen et al. [40] showed that diagnostic evaluation is a powerful tool for uncovering text mining performance problems that are not revealed by the standard paradigm of calculating F-measure on a corpus, i.e. performance evaluation. They ran five entity identification systems against a synthetic test set designed to explore linguistic aspects of the GM input space. This form of testing identified a variety of undocumented and unsuspected problems in the systems under test. Such diagnostic evaluation is demonstrably valuable; so is global performance evaluation via the standard metrics in shared community challenge tasks. We show in this paper that there is still more insight to be gained into text mining tools than either of these paradigms provide.

Discovering insights into these systems is a complicated task. Adoption of UIMA is not trivial – it is not a lightweight architecture, and it requires considerable software engineering abilities. Despite these costs, the use of UIMA in general, and this evaluation platform in particular, can provide gains in efficiency over time for the NLP community as a whole if it is adopted by the community at large. By necessitating a standardized interface between components the framework inherently promotes the sharing of NLP tools and eases the workload typically involved with integrating third-party software. It is likely that as time passes, if the framework is adopted by our community, it will become progressively easier to combine various language processing components that have been released publicly. Our initial download serves as a starting point for this process. We have included most components used in the example evaluations discussed in this paper.

Systematic understanding of the causes of performance differentials, particularly those that involve interactions among subtasks or between processes and particular classes of text, is necessary to reach the performance required for text mining to have a substantial impact on biomedical research. To achieve this understanding, a robust architecture for performing large-scale, flexible evaluations is essential. The code base demonstrated in this paper and made freely available on SourceForge.net is such an architecture. The BioCreative organizers are currently attempting to build a similar platform for evaluation of text mining systems on the BioCreative 2006 tasks. We have contributed to that effort and are also collaborating with the UK National Centre for Text Mining and the University of Tokyo Tsujii Lab. to develop a web-based interface to an architecture similar to the one described in this paper [41]. Both of these efforts underscore the significance of the work reported here.

## Conclusion

Scientific collaboration is hindered by disparities in data formats at multiple levels – minimally, those of inputs and of outputs. Conversely, collaboration is facilitated when such disparities can be factored away. One of the significance claims for this work comes from the ability of the software artifacts that we have released to facilitate collaboration by enabling a common interface between systems with otherwise disparate input requirements and output formats. It has already enabled collaborations between our group and groups in the US, Japan, and the United Kingdom, and work is underway to construct publicly available interfaces to similar systems in Europe and Japan. The potential for this architecture to facilitate both discovery and collaboration has only barely begun to be realized.

## Availability and requirements

• **Project name: **BioNLP-UIMA Component Repository

• **Project home page: **

• **Operating system(s): **Platform Independent

• **Programming language: **Java

• **Other requirements: **Java 1.5 or higher

• **License: **GNU GPL v2.0

• **Any restrictions to use by non-academics: **None

## Abbreviations

GM, gene mention; GN, gene normalization; NLP, Natural Language Processing; BioNLP, Biomedical Natural Language Processing

## Competing interests

The author(s) declare that they have no competing interests.

## Authors' contributions

WAB designed and conducted the experiments and implemented the code base for the evaluation platform. KBC supervised the project. LH conceived the original concept. WAB and KBC drafted the manuscript. All authors approved the manuscript.

## Supplementary Material

Additional file 1 Evaluation platform source code. The additional file contains the software evaluation framework discussed in this paper. The most current release can be downloaded from SourceForge.net [1]. See the accompanying README file for instructions on installation and use. Also included in the distribution is a collection of UIMA wrappers for some commonly used BioNLP tools and annotated corpora. Click here for file
